# “I6 passages: on the reproduction of a human embryonic stem cell line from Israel to France”

**DOI:** 10.1080/14636778.2018.1548269

**Published:** 2018-12-02

**Authors:** Noémie Merleau-Ponty, Sigrid Vertommen, Michel Pucéat

**Affiliations:** a Department of Sociology, University of Cambridge, Cambridge, UK; b Department of Global Health & Social Medicine, King's College London, London, UK; c Institut National de la Santé Et de la Recherche Médicale U1251, Aix-Marseille University, Medical School La Timone, Marseille, France

**Keywords:** stem cells, biobank, France, Israel, passage, reproduction

## Abstract

The first French clinical trial using human embryonic stem cells for regenerative purposes was launched in 2014, using the I6 stem cell line that was imported from Israel. From Israel to France, national reproductive policies and practices inform how basic scientists produce, manage and circulate cells across countries. Building on an interdisciplinary co-production involving two social scientists and a life scientist, this article suggests that biobanks *passage* cells from in vitro fertilization to stem cell science and from country to country by modifying their reproductive meaning. Four passages are described: the absence of cells in 2005 when the research started in France; the presence of supernumerary embryos available for research in Israeli IVF biobanks; the production of the I6 stem cell bank in Israel; the importation and laboratory biobanking of the cells in France. Human embryonic stem cell lines can never be completely disentangled from reproduction.

## Introduction

In 2007, the biology journal *Stem Cells* published an article which opened the path towards the first phase one clinical trial using huES in France to treat infarcted hearts (Tomescot *et al*. 2007). When the trial was finally launched on the 21 October 2014 at Georges Pompidou Hospital in Paris, the media^1^ reported on the story by focusing on the achievement of the French scientific teams who successfully tested this new protocol on a patient after the production of an embryonic stem cell bank.^2^ In the press release written by the public research institute that conducted the basic research, cells are referred to generically as “human embryonic stem cells,” with no more information. This release stresses the involvement of French biologists and French surgeons working for public institutions.

In a country where research on human embryonic stem cells has been completely banned until 2005 and is still tied to sensitive bioethical debates on human embryos and reproduction, this clinical trial can be seen as the realization of a “national ambition” (Sleeboom-Faulkner 2011). This national ambition seems to have been able to hold together scientific excellence and strong national bioethics. However, cells are never generic. They belong to specific lines, in this case the I6 stem cell line, which was developed at the Technion Institute of Technology in Israel. This international scientific collaboration complicates the French national narrative. It also shows that biobanks are central to not only the production and international exchange of scientific materials, but also to their nationalization. This invites us to discuss anthropological and STS accounts of the links between reproduction and basic science.

In the circulation^3^ of cell lines through biobanks “repronational histories,” referring to the ways in which national identities and reproductive politics constitute each other (Yuval-Davis 1998; Franklin and Inhorn 2016; Roux and Courduriès 2017),^4^ are central to the emergence of the regenerative medicine field. This suggests that internationally circulated human embryonic stem cell lines are not entirely “disentangled” from their local provenances, and their reproductive contexts (Gottweiss, Salter, and Waldby 2009, 35). Inspired by the notion of “co-production” (Jasanoff 2004), this article resulted from a collaboration between two social scientists and a life scientist. It aims to underline that even when scientific materials such as human embryonic cells are disentangled from personal reproductive histories, they are still tied up to reproduction at a national level. We call *passages* the processes through which human embryonic stem cells travel across countries and institutions. The term “passage” refers to splitting cells from one dish to several dishes in order to amplify their number. It also has a history in STS (Callon 1986), and we repurposed it to describe another kind of use and amplification of cells.

After presenting the methods that were used in this collaborative article, we discuss the notion of disentanglement in stem cell derivation from human embryos. Then, we underline four *passages* relating to the co-production of the I6 line through biobanking circulation. Starting from the French project of huES cardiac differentiation, we delineate why the team decided to rely on an international network to import the cells. When the project started in 2005, just after huES research was authorized under exceptional circumstances, there was no biobank in France. This is due to a national framework that values reproduction over basic science and regulates the latter within the understandings of the former. The second passage implies the presence of numerous supernumerary IVF embryos biobanks in Israel which has co-produced a very intimate “IVF – stem cell interface” (Franklin 2006). The third passage concerns the storage of the I6 in an Israeli research facility that is strongly connected to international scientific networks as providers of resources and technologically innovative expertise. Finally, the fourth passage involves the biobanking of the I6 line in the basic science French laboratory, after they had been imported. As they cross borders from Israel to France, cells are nationalized through a specific French regulatory biobanking regime. In these biobanking practices, cells are *passaged* in the sense that their use is amplified while their reproductive meaning is multiplied by circulating from space to space.

## Methods

This article results from the multidisciplinary collaboration between two social scientists and a life scientist. Our collaboration explores the ways in which researchers across disciplines can tackle shared problems, while not reducing our topic to one view or the other. Noémie Merleau-Ponty is a sociocultural anthropologist who researches on IVF and stem cell research in France. Her contribution to this article is based on her doctoral research conducted at the School of Advanced Studies in the Social Sciences and on the collection and analysis of primary data since 2011 (French legislation to be found on the national website “legifrance.fr,” institutional documentations such as reports from the national committee of bioethics – Comité consultatif national d’éthique – and the National Biomedical Agency's authorization forms of huES cell importation, 22 interviews with actors of the IVF – stem cell interface in France, as well as participant observations in basic science facilities between 2011 and 2013). Unlike the Israeli stem cell sector, the French scientific stem cell context is still understudied from an anthropological perspective, which explains the difference in references with the Israeli case studied by Sigrid Vertommen.

Sigrid Vertommen is a historian and political scientist who conducts fieldwork-based research on the political economy of assisted reproduction in Israel/Palestine. Her contribution to the article is informed through 99 semi-structured interviews with the various stakeholders who have shaped Israel's stem cell policies and practices (including stem cell scientists, embryologists, fertility doctors, representatives of the Israeli Ministry of Health, members of the National Bioethics Committee, rabbis, representatives of feminist organizations, CEOs from stem cell companies and technology transfer companies) and through a close reading of institutional documents by the National Bioethics Committee, the Israeli Academy of Sciences and Humanities and the Ministry of Health. This data collection largely took place in the context of her doctoral research at Ghent University in Belgium, however, additional interviews were conducted with the Israeli stem cell researcher who was involved in the production of the I6 stem cell line.

Michel Pucéat is a life scientist, a developmental biologist, who has been involved in the stem cell field since the authorization to use HUES cells in France in 2005. The laboratory he runs has used the stem cell line that is the topic of study in this article. His contribution to this article is based on his involvement in stem cell research nationally and internationally. He has given access to primary data, continuously discussed them with Noémie Merleau-Ponty and Sigrid Vertommen and shared information as well as has participated to the writing of this article.

The two social scientists follow what Choy *et al*. (2009, 380) have stressed when writing on the Matsutake mushroom: “cultural anthropologists’ interests in global and multisided phenomena require new kinds of ethnographic methods.” The two social scientists are collaborating based on their individual fieldwork experiences in France (N. Merleau-Ponty) and Israel/Palestine (Sigrid Vertomment). The collaboration between them and Michel Pucéat is rooted in the conviction that if we are to take the co-production idiom seriously and its “self-conscious desire to avoid both social and technoscientific determinism in S&TS accounts of the world” (Jasanoff 2004, 20), then we need to implement it in our methods, as well as in our analytical perspectives.

## Passages of cells through sites of conservation (biobanks)

The main argument of this article is that the making of the I6 line and its international travel from Israel to France is co-produced (Jasanoff 2004) by *passages* through several biobanks that may entangle or disentangle (Gottweiss, Salter, and Waldby 2009) repronational contexts (Franklin and Inhorn 2016).

### Co-production of basic science and reproduction

The different steps involved in the production of the I6 line from an IVF embryo and its storage in various biobanks and countries entail several types of “exchange regimes” (Milanovic, Pontille, and Cambon-Thomsen 2007) – biological, spatial, legal – all informed by repronational traits. In *States of Knowledges,* Jasanoff (2004) introduces the well-known idiom of co-production to explain how science and society constitute each other without giving primacy to one or the other. Her proposal stems from a concern that much of the existing research on science and technology “has not sought to build systematic connections between the micro-worlds of scientific practice and the macro-categories of political and social thought” (*Id.*, 18). She continues her critique that

generally, STS work has been less successful than political science in finding places for human beliefs and imagination, and in accounting for significant economic, technological and social disparities in the practices of world-making; nor has science studies paid much attention to what happens when particular epistemic and material constructions of the world circulate through societies configured by very different historical and material constraints. (Jasanoff 2004, 28)

These suggestions inspire our study of how the I6 line is made and circulated through global scientific practices within repronational contexts.

Local contexts of human embryonic stem cell science, as underlined by Hauskeller and Weber (2011), have been extensively studied. Writing on induced pluripotent stem cells, they indicate that most of the social science work done on stem cells highlights the specificities of local contexts, while their article focuses on a more global scale of analysis, showing the existence of an international community of scientists. Gottweiss, Salter, and Waldby (2009) have also written about the global politics of human embryonic stem cell science and suggested that it involves a process of disentanglement from reproduction and local contexts:

The hESC line is a highly *disentangled* entity compared with the IVF embryo from which it was derived. Whereas the IVF embryo is caught up in a dense web of reproduction, family relations and social controversy, a gradual process of purification – donation to clinic and then to laboratory, disaggregation, immortalization, passage – transforms the embryo into a more properly anonymized, scientific object. It becomes a neutral, validated entity, denatured of its local significance. (Gottweiss, Salter, and Waldby 2009, 35)

An institutional process of disconnection and re-identification accompanies the derivation of cells. The I6 cells have been detached from the original embryo through an anonymous gift to research, derivation into a cell line, and renaming. Naming a cell line after an institution following its anonymization from patients tends to assert this disentanglement. However, saying that these cells no longer have a history because of the erasure of their past and the production of a narrative of futurity (Glasner 2005), tells only one side of the story. Franklin (2006) already noted how, despite its universalizing and totalizing claims, the field of reproductive biomedicine and stem cell research is also defined by its localized character as it always depends on a particular context of clinical practice (such as the willingness of couples to donate their reproductive tissues), ethical regulation (concerning the status of the human embryo, patient information, informed consent) and a specific spatio-temporal “fix” of biocapital. These particularities are of course as much local as they are global, as they are rooted in ongoing histories and global geographies of science, colonialism, and capitalist modernity. They are particularly clear in IVF-stem cell interfaces in which

the ‘double reproductive value’ underlying huES cell derivation explores the meaning and sociological implications of the reproductive value of stem cells ‘in themselves’ as *both can and cannot be separated* from their reproductive value in the context of assisted reproduction. (Franklin 2006, 85)

### Passages

The meanings of the word “passages” in this article are multiple. In biology, a passage of cells is the process of splitting them from one dish to several dishes in order to amplify their number. This is a mandatory process for huES cells to grow and this is the meaning implied by Gottweis, Salter and Waldby (*ibid.*). For the I6 cell line in Israel, this process started right after the derivation of the line from the embryo. Then, after their transfer to France, the French scientists kept growing the cells and continued passaging them. Cells can thus prolong their life and be expanded as a stock, and they can be available for multiple usages. Cells are grown in a medium and some of them are frozen and stored for future uses, while others are experimented on by adding reagents that interact with them. Frozen vials can be shipped to different laboratories and countries through “Material Transfer Agreements” (MTA) that have different contents depending on the institutions and the various regulations implied. The notion of “passage” also exists in Science and Technology Studies.

In his article on the sociology of translation, Callon (1986) introduced another view on “passage.” In this text, he investigates how science and technology structure power relationships in the domestication of scallops in St Brieuc Bay. He analyzes how three researchers intervene in St Brieuc, where a controversy erupted around the population decline of scallops. The researchers manifest their power in the networks at play by defining “obligatory points of passage” that can fulfill the hypothetical wishes of different actors (scallops’ survival, fishermen's income and scientists’ knowledge) (Callon 1986, 205–206). Similarly, the meaning of “passage” in this article highlights how different actors with different identities and interests connect to one another (both nationally and internationally).

By focusing on biobanks as sites through which human embryos and human embryonic stem cells are cryopreserved and shipped across the world, we suggest that the co-production of repronational frameworks and the I6 line materializes through four “passages.” Furthermore, we also suggest that French and Israeli passages are connected through international exchanges, even if their logics are very different; and, that French biobanking practices are enabled by Israeli passages that are nonetheless silenced when the cells are imported into French settings.

## The French IVF/stem cells interface: no cells to passage in 2005

Human embryonic stem cells are undifferentiated cells extracted from blastocysts (day 5 or 6 embryos). The derivation and cell culture of stem cell lines is the foundation for the production of materials for bioscientific laboratories specialized in stem cell research. In 1998, the first human embryonic stem cell line was derived in the USA using an embryo from and in collaboration with Israel (Thomson *et al*. 1998). The 2000s saw a new research field emerge from this starting point. Depending on the country, legislations were more or less prone to support this type of research. In France however, research involving huES was forbidden until 2005. From 2013, research was only allowed when specific authorizations were given by the National Biomedical Agency. In 2007, Tomescot and his colleagues published an experimental work that had started two years earlier when French scientists obtained the official authorization to use, but not yet to derive, human embryonic stem cells in their research (revision of Bioethic law n° 2004-800 from 6 August 2004 relative to bioethics). In France, huES regulation is organized under the broader category of research on human embryos, and is strongly connected to assisted reproductive technologies. Although huES are not embryos and leave the “reproductive trail” after their derivation into stem cell lines (Thompson 2005), they are administered through its bioethical framework, resulting in a distinctive tension: huES science is viewed as dangerous for human embryos because it considers cells as mere materials. Memmi’s (1996) work analyzed how French ethical considerations are imbued with reflections on human bodies’ frailty, their biomedical objectification as well as on the need for cures. The human body is both defined as a “taboo” and an “instrument” (*ibid*., 28). Similarly, human embryos are defined in a lose way, which allows different actors to have different interpretations. In legal and bioethical texts, Raschini (2012, 58) analyzed the use of “categories of approximation” (*approximation catégorielle*) that leave spatial and temporal frontiers of meaning unlimited or open for transformation, such as the notion of “potential human person.” Indeed, in France, huES science is regulated through a legal, bioethical and public reproductive framework that views embryos as neither complete objects of science nor complete subjects of humanity (Merleau-Ponty 2018).

As it is forbidden to create human embryos for research purposes only (article L2151-5 of Public Health Code), the cells come from reproductive clinics where patients keep frozen supernumerary embryos they can give to research. These embryos are protected by a set of regulations stemming from a tradition of bioethical discussions as old as the first French birth resulting from IVF in 1982. Indeed, the French National Consultative Bioethics Committee (CCNE) was created in 1983 by President François Mitterrand. As described through the committee's website, it aims at “elucidating scientific progress, raising new issues challenging society and observing change from the perspective of ethics.” Its first recommendation (CCNE 1984) addressed reflections on embryos and fetuses and their status as “potential human person.” “This human quality,” the text says, “puts an obligation on researchers and therapists to respect the embryo. Only the manner in which this respect is expressed may vary according to the aims pursued.” (CCNE 1984, 3–4) This “human quality,” continues the recommendation, protects the embryo against commercial or industrial uses. This exceptionality of the “human,” which imbues these cells with a status that is more than just biological, has been kept throughout time and debates, as proven by this extract of a 2010 CCNE recommendation, which recapitulates 26 years of debate and stresses that:

If the embryo *in vitro*, as soon as it is created, is considered to be a person already, the ethical issue of the creation of spare embryos and therefore of their possible destruction, does not even arise: creating spare embryos is unacceptable. If, on the contrary, the embryo in vitro is seen as nothing more than a bundle of cells, the ethical issue of creating embryos for research purposes does not arise either: creating embryos for research is not a problem. Stating that the potential human being is an enigma means that, after hearing out these two extreme and mutually exclusive positions, however justified they might be in principle, one chooses to adopt an attitude which can truly cope with this difficult and essential in between concept: a ‘potential human being’. (CCNE 2010, 10–11)

This in-between status consistently re-appears in French bioethics (Jouannet and Paley-Vincent 2009). As Bateman (2009, 105–6, my translation) summarizes:

The question ‘who or what?’ suggests that the discussions on the governance of embryos are understood as an ontological debate: is the embryo a thing that can be disposed of like any other human tissue, or is it a full person, endowed with full rights? (…) However, this debate, which is old and more complex than the ‘who or what’ question, is far from being resolved and might never come to a satisfying solution for all.

The Second World War and the Nazi machinery of eugenics and extermination has profoundly oriented French bioethics towards protecting nascent human lives (Memmi 1996; Rabinow 1999). This protective status can also be explained by the political power of the dominant religion in the country. Christianity, especially Catholicism, is well represented in France, and its pro-life and nature arguments have been addressed and analyzed by social scientists (Bateman 2002; Mathieu 2013; Merchant 2014). However, this protective status does not consider embryos to be full-fledged persons, which makes sense in a country where abortion rights are strong (Boltanski 2004). Since 1975, when abortion was legalized in France, the Veil law allows women access to “voluntary termination of pregnancy” up to twelve weeks of gestation. The medical procedure is free and publicly funded.

Taking all these ethical and reproductive considerations on the potential personhood of human embryos into account, France first banned research on human embryos or their cells. In the late 1990s and early 2000s, French scientists could not derive cell lines from IVF embryos or import any of them from abroad, as the National Biomedical Agency would not authorize such procedures. In 2005, the ban on huES research was smoothed to support research with therapeutic goals, however, due to the previous legislation, no stem cell lines were available in the country. Line derivation is not an easy task and requires significant human and financial resources. Line derivation is a project which the French team who published the cardiac differentiation protocol in 2007 had no intention to pursue. The research was authorized because of its therapeutic goal, not because it was meant to derive cell lines. Relying on international connections, Israel was one of the few countries where line derivations had been developed during previous years. Why specifically this country? This is where the best expertise had been set. Trying to understand what enabled this expertise opens a wide range of topics that cannot be reduced to technical skills only. By focusing on two passages in Israeli biobanks, i.e. the abundant presence of supernumerary IVF embryos and their availability for research purposes as well as the production and storage of the I6 line, we unpack the particularity of Israel's reproductive-embryonic interface.

## IVF bank: the Israeli IVF-stem cell interface at the frontier

The I6 stem cell line was derived in 2000 at the Technion, Israel's prestigious Institute of Technology, by Professor Joseph Itskovitz-Eldor and Dr. Michal Amit. Just a few years earlier, in 1998, both stem cell researchers collaborated with James Thomson's team in Wisconsin to develop the first human embryonic stem cell lines. As Michal Amit recalled of this collaboration (interview Haifa, 27/07/2016, our emphasis):

I spent a few months in Wisconsin to learn about the derivation techniques, and then I came back to Israel. We used five frozen embryos, six days old blastocysts that were left over from fertility treatment, only three resulted in a successful derivation, the I3, I4 and I6 lines. These were the first **home-grown** Israeli stem cell lines. Benjamin Reubinoff may have been the first Israeli researcher to establish stem cell lines in 2000, but he did it abroad with Alan Trounson and his colleagues at Monash University in Melbourne, and then **returned them back** to Israel.

Being home-grown or native-born is an important reference in Israeli society, which in colloquial Hebrew is proudly referred to as being a “Sabra.” A Sabra is a Jew who is born on Israeli territory rather than in diasporic exile. The term, referring to the cactus fruit that is known to be tough and prickly on the outside and sweet on the inside, first appeared in the 1930s and was used by the Zionist movement to celebrate the “New Jew” that emerged in Mandatory Palestine. Unlike the diasporic “Old Jew” who was often depicted as weak and powerless in Zionist discourse, the “New Jew” was portrayed as a fighter, a kibbutz member or farmer who showed strong, healthy and productivist physical qualities (Almog 2000; Weiss 2002; Davidovitch and Seidelman 2004).

The I6 “Sabra” stem cell line constitutes an interesting entry point to understand the particularity of Israel's reproductive-embryonic sector, which for over two decades has been internationally acclaimed for its scientific excellence and commercial success. Since the field of stem cell research was established in 1998, Israeli scientists such as Benyamin Reubinoff, Joseph Itskovitz-Eldor, Michal Amit, Karl Skorecki, Dalit Ben-Yosef and Nissim Benvenisty have been at its “frontier” (Vogel 2002; Barilan and Siegal 2004; Prainsack 2006; Simonstein 2008; Birenbaum-Carmeli and Carmeli 2010; Ben-Or and Ravitsky 2010; Shalev and Hashiloni-Dolev 2011; Vertommen 2017). In 2002, Science Magazine lauded Israel as one of the top countries in stem cell research (UKSCI 2005). The first round of publications on human embryonic stem cells in the late nineties almost all included Israeli authors and the first two research teams that succeeded in isolating human embryonic stem cell lines both involved Israeli researchers (Vogel 2002). Since then, Israel's stem cell field has been advancing a broad array of discoveries, including the development of xeno-free and defined growth media, cell culturing scale-up methods, genetic manipulation techniques and protocols for induced differentiation of the cells into desired cell types.

These scientific developments have taken place in one of the world's most lenient regulatory frameworks concerning embryo research. In 1999, Israeli law makers adopted the “Ban of Genetic Intervention Law,” which put a moratorium on germ-line genome modification and reproductive cloning in humans that is renegotiated after every five years, while explicitly allowing the use of human embryos for stem cell research and the production of cloned blastocysts for generating human tissues (Revel 2000; Israeli Academy of Sciences and Humanities 2001). Israel's “fearless, liberal but not immoral embrace of stem cell research,” as Prainsack and Firestine (2006, 42) summarized, has been traced back to Judaism's tolerant stance on techno-scientific interventions (Simonstein 2008; Ben-Or and Ravitsky 2010). Unlike Christianity, Judaism does not oppose human embryonic stem cell research. Talmudic tradition dictates that life does not begin at the moment of fertilization, but gradually takes shape after a period of post-implantation development. Before the 40th day of fertilization an embryo is considered to be *maya b’alma* or mere water (Steinberg 2003). As Michal Amit (interview Haifa, 22/02/2012) clarified:

According to Jewish religion an embryo only becomes a person with rights and a soul after 40 days of fertilization. The five days old embryos that we used for the derivation of the I6 cell line were not seen as human beings, and we were not perceived as if we were executing living persons by using these embryos for science.

Judaism and Jewish law also attach chief importance to healing and saving lives, which allows for human interventions into God's creation. As one famous reproductive biologist from Hadassah Hospital phrased it (interview Jerusalem, 04/03/2012):

In the Jewish ethos life precedes every law. There is no law that is stronger than life itself. This is an important ethos. […] Here in Israel life supersedes everything else. People would go a long way in order to preserve life, to give life, to help cure. So life has a different meaning here.

Rather than being antagonistic forces, Israel's religious and scientific communities have thus forged a remarkable alliance in its policy views and recommendations on embryonic research (Nahman 2013).

Although Jewish religious prescriptions are obviously crucial in understanding Israel's lenient stance on cloning and embryo research, they do not suffice to explain all. As Prainsack (2006, 189–190) rightfully noted: “Religion alone tends to explain too much, and therefore it explains too little.” She referred to the secular segment of Israel's population that is as permissive in its attitudes toward embryo research and cloning as its religious counterpart. In addition, she suggested that whereas Israel's regulations on this matter takes explicit reference to Jewish law, in other biomedical matters this is not always the case. In other words, rather than strictly focusing on cultural and religious narratives of “Jewishness” to understand the production of the I6 cell line, other socio-political entanglements should also be considered. This section analyzes the production and circulation of the I6 stem cell line within and across Israel's repronational “frontiers” by taking into account Zionism's demographic aspirations and Israel's leading position in the global bio-economy (Vertommen 2017).

The I6 stem cell line was derived from one of the tens of thousands spare embryos that are stored in the freezers of Israeli fertility clinics. Although Israel's Ministry of Health is not keen to publicize the exact numbers, it is a well-known fact that there are large amounts of supernumerary frozen embryos from fertility treatments in Israeli IVF banks (Raz *et al*. 2016). According to Israeli guidelines spare embryos are to be stored for free for five years after which the patients need to make an embryo disposition decision. Patients can either pay to keep the frozen embryos in storage, donate the embryos for medical research, or thaw and destroy the embryos (Israeli Public Health Regulations on In Vitro Fertilization 1987 and MOH circular Guidelines for patients on use of frozen fertilized oocytes in in vitro fertilization units 2008). It was the availability of large amounts of spare reproductive tissues and the initial willingness of Israeli couples to donate their embryos for research purposes, which enabled the early stem cell research discoveries in Israel.^5^

When asking Michal Amit about the ground-breaking role of Israel in stem cell research, she replied:

There are “two reasons for Israel's success: first, the good connections of Professor Itskovitz on a friendly and scientific level with James Thomson, and the fact that in Israel you have a lot of surplus embryos. Israel is one of the leading countries of IVF cycles. So if you do a lot of IVF, you have a lot of surplus embryos that nobody wants”. (interview Haifa, 22/02/2012)

The coordinator of the Women and Medical Technologies Program of the Israeli feminist organisation Isha L’Isha, added (interview Haifa, 28/01/2012):

There is kind of a joke among researchers. They say that most of the eggs and embryos that are circulating in global stem cell research around the world are coming from IVF clinics in Haifa.

Four of the five original cell lines that were developed in 1998 by Thomson and his research team were created out of spare IVF embryos from the fertility clinic in Rambam Medical Center in Haifa, headed by Joseph Itskovitz-Eldor (Thomson et al. 1998, 1145). Via Dr. Michal Amit, more than a dozen frozen embryos donated by Israeli couples reached Thomson's lab in Wisconsin (Vogel 2002).

In Israel, the IVF-stem cell interface has always been very outspoken and osmotic. Most of Israel's ground-breaking work in stem cell research has been conducted by fertility specialists who enjoyed a decennia-long, solid experience in IVF, a reproductive technology that is not only abundantly used, but also fully subsidized by Israel's pronatalist fertility regime. In an interview with Science Magazine, the Israeli stem cell scientist Reubinoff (quoted in Vogel 2002, 1819) remarked:

Given the importance of IVF expertise and connections to the research, it isn't surprising that Israeli researchers were involved. According to Jewish tradition, to procreate is very important and there is a lot of support for infertility treatments and a very large number of IVF clinics in Israel*.*

Israel has one of the world's highest number of fertility clinics per capita, and measured by the number of IVF treatment cycles per capita, Israelis are by far the biggest consumers of IVF in the world (Sullivan *et al*. 2013; Birenbaum-Carmeli 2016).^6^ Assisted reproductive technologies such as IVF, intracytoplasmic sperm injection (ICSI), surrogacy, egg donation, egg freezing and preimplantation genetic diagnosis (PGD) are not only immensely popular and widely accepted in Israeli society, but most of them are also generously subsidized by the State (Shalev and Werner-Felmayer 2012; Gooldin 2013). For instance, the Israeli government provides every citizen of the country with an unlimited number of IVF cycles until the live births of two children within the current relationship (Birenbaum-Carmeli 2004). This pronatalist stance is not only rooted in cultural and religious commandments of “being fruitful and multiply,” but also in Zionist anxieties on how to guarantee a Jewish majority in a Jewish State without being demographically outnumbered by native Palestinians (Portuguese 1998; Kanaaneh 2002; Nahman 2013; Vertommen 2016). As former Minister of Health Mordechai Gur commented, when asked about Israel's exceptionally generous IVF policy: “IVF is still cheaper than a newcomer” as a means of increasing the size of the population (quoted in Birenbaum-Carmeli 2004, 900).

Zionism's core ambition to create and consolidate a Jewish homeland in Israel/Palestine has not only co-produced a pronatalist demographic logic, but – related to this – a considerable belief and trust in the therapeutic and diagnostic powers of medical science and technology. Without them, the Zionist promise to turn the presumed barren desert into fertile land and bodies could not have been upheld (Prainsack 2007). Similar to other colonial endeavors, the Zionist movement has used medical science and technology to legitimize the colonial settlement of what was depicted as an empty, backward and disease-ridden land (Efron 2007). Apart from providing scientific legitimacy to the Zionist project, these technologies were also required for the creation of a healthy and vitalized Jewish nation after centuries of collective extermination and what was seen as diasporic decline and degeneration. (Davidovitch and Seidelman 2004; Efron 2007). Sufian (2006, 390) for instance, described how Zionist scientists in the early twentieth century “used malariology to construct intellectual, scientific borders that mobilize the ‘the nation’ while improving its health.” Birenbaum-Carmeli and Carmeli (2010) and Vertommen (2017) reached similar conclusions on Israel's scientific research on human fertility and stem cells that is often described as “pioneering” or “frontier science.”

Particularly in a context as Israel/Palestine that is increasingly framed as a settler colonial one (Yuval-Davis and Stasiulis 1995; Veracini 2006; Wolfe 2007, 2016; Jabary-Salamanca *et al*. 2012; Shalhoub-Kevorkian 2015; Lentin 2018), it is worth unpacking how such metaphors are deployed to describe national imaginaries on science and technology. Ceccarelli’s (2013) research on the embeddedness of the frontier metaphor in American scientific rhetoric described how the term evokes an agenda of “science as a contest of territory, always on the edge and on the verge of new discoveries.” What defines the frontier are boundaries that are never fixed and stable, but always on the verge of being crossed, expanded, explored, conquered and tamed. While the frontier myth has been glorified as the essence of America's success story, it has been rightly criticized in postcolonial and indigenous STS studies as the source of conquest, exploitation and extermination of native and racialized populations (Anderson 2002; Harding 2011; Hinterberger 2013; TallBear 2013; Pollock and Subramaniam 2016). Israel's scientific frontier mentality can be best captured by the Hebrew term “*chutzpah*,” which Senor and Singer (2011, 30), referring back to Leo Rosten's “The Joys of Yiddish” (1968), described as “a gall, brazen nerve, effrontery, incredible guts, the presumption plus arrogance, such as no other word and no other language can do justice to.” In their popularized analysis of the techno-scientific and commercial success of Israel's “start-up nation,” Senor and Singer (2011, 106) also refer to the term *bitzu’ism,* as “a kind of pragmatism that is at the heart of the pioneering ethos and Israel's entrepreneurial drive.”

This “pioneering” ethos is also present in Israeli discourses and practices of human embryonic stem cell research, where the belief in medical and scientific progress tends to overhaul fears of bioethical and societal dystopias. For instance, when asking one Israeli stem cell researcher in what kind of legal climate the early stem cell discoveries took place in Israel, she replied (interview Jerusalem, 18/07/2012):

When the first stem cell researchers got involved there was not really a strict legislation. You know, once things were working and many labs were beginning to derive and many centers were beginning with derivation, there was a need for legislation. Yet in the beginning, there was science before legislation.

While in France such embryonic experimentation would not have been possible, Israel's frontier imaginaries did not obstruct this technoscientific practice. Rather than referring to Jewish or Israeli character idiosyncrasies, such as *chutzpah* or *bitzu’ism* to explain this, it could be useful to understand it from a (settler)colonial perspective.

## The I6 bank between bioethical leniency and academic entrepreneurialism

After its configuration, the I6 cell line was stored in the WiCell stem cell laboratories in Wisconsin, where they fell under the regulation and ownership of T3, the technology transfer company of the Technion. Michal Amit recalled (interview Haifa, 27/07/2016):

In those days it was the Technion that approved or disapproved the movement of the I6 line. The material transfer agreements at the Technion are not very restrictive, compared to other research institutions. This is because we want the knowledge and materials to be used by other institutions, so that they can further develop.

Unlike in many other countries where the export of stem cell lines is decided on a national level, in Israel this decision is made by the individual research institute without public discussion or the involvement of national committees on medical ethics. As Itskovitz and Amit were hopeful and determined to quickly develop clinical applications in the field of cell repair, they were also keen to export Israeli stem cells for biomedical research by cooperating with stem cell laboratories in other countries (Gross 2001, 582). In the early 2000s the Technion was therefore one of the few centers in the world where stem cell researchers from more restrictive countries “would do their shopping” (Gross 2003, 244). In the case of the I6 cell line, this led to several controversies. In 2003, for instance, the stem cell line was exported to Bonn University in Germany for research on nerve cells causing moral criticism. The German reluctance to allow research with huES cell lines was rooted in Kantian views on man as an end in himself, and in painful collective memories of Nazi experiments on human beings. This resulted in remarkable discussions on the morality of human embryonic stem cell research in which Israeli bioethicists urged their German counterparts for calm and moderation. The Israeli bioethics expert Asa Kasher told the newspaper (Ha’aretz, 09/01/2003): “Perhaps we, the Jews of Israel, are the only ones who can tell [the Germans] them that on this matter, they are exaggerating.”

Today, the I6 cell line is still property of the Technion, but the exclusive license is with Accellta, a spin off company from the Technion. Accellta was founded in 2012 by Amit and Itskovitz to provide customized solutions for mass production of human embryonic and induced pluripotent stem cells for drug discovery, regenerative medicine and research. Since then, the export of the I6 line has become costlier. While in 2005 the French team from INSERM could still import the cell line without any cost except the shipping costs, this has changed since 2012. Michal Amit (interview Haifa, 27/07/2016) remarked:

Last time, few months ago when I had to arrange an MTA for the cells, the price was 1300 dollars plus shipping costs, which is really not that much if you come to think of all the tender love and care and money that we have put in deriving these cells.

The stem cell sector constitutes a key sector in Israel's bio-economy that since the late seventies has been positioning itself as a major player in the global market of biomedical research and development, with a special focus on healthcare (Prainsack and Firestine 2006; Vertommen 2017). Accelta is one of Israel's 18 biotech companies that are currently developing and marketing cell-based regenerative therapies, with Pluristem, Cell Cure, Gamida Cell, Brainstorm and Kamidastem being the most promising ones. Most of Israel's stem cell companies are former start-up companies from Israeli universities and research centres that are known to be strongly oriented towards the market. Messer-Yaron (2011, 17) stated that Israeli research institutions were among the first in the world to commercialize their academic discoveries through the successful creation of technology transfer companies, prompting her to term Israel not the only, but “by all measures- a best practice example” in matters of technology transfer. Israeli tech transfer companies, such as T3 from the Technion are among the world's top in terms of revenues and patent holders in the field of medical devices and bio-pharmaceuticals (Israel’s Foreign Investments and Industrial Cooperation Authority 2016). From the early stages in the research process, Israeli researchers are urged to commercialize their knowledge production through the creation of patents or spin offs (Oliver 2004).

To understand the production of the I6 line one needs to understand the particularity of Israel's repronational IVF-stem cell interface. Similarly, the reception of these cells in France is not only about importing a global material, but also nationalizing it.

## Nationalizing the resource: authorization of importation and lab biobanking

What happens when French biologists ask for an authorization to import huES from abroad? They have to fill in a lot of documentation for the National Biomedical Agency to verify the legitimacy of the demand. The National Biomedical Agency asks whether the lines are registered at the UK Stem Cell Bank or the National Institutes of Health in the USA (NIH). These two institutions play a powerful and active role in standardizing practices, through which a bioscientific community works for the promotion of a new field of research (Webster and Eriksson 2008; Eriksson and Webster 2015).

The importation of cell lines is also subject to national criteria that have nothing to do with standardization of global materials. A major therapeutic progress must be underlined, and research strictly oriented towards the widening of knowledge is not authorized (article L2151-5 of the Public Health Code). Furthermore, article 16 and 16-8 of the Civil Code must be respected, stating the “primacy of the person,” forbidding any “violation of its dignity” and “guaranteeing respect for the human being from the beginning of its life.” Regarding *in vitro* human embryos, these elements point to the “anonymous” and “free gift” to research from “a couple” or “the surviving member of this couple” who gave a “consent” after having abandoned their “parental project” and after having been informed of other options, like gift to another couple or destruction. How can the National Biomedical Agency verify if all these criteria have been respected? Only some of the criteria can be documented, such as the therapeutic goal, the consent, the anonymity and the free gift.

The clauses on the “primacy of the person,” its “dignity” or the “beginning of life,” however, are more difficult to verify. These notions relate to a specific French framework that we addressed at the beginning of this article. The logic behind the notion of “potential human person” sustained an open but protective view on human embryos. However, this notion depends on the national context, for example, if we think of the United Kingdom where research is authorized until 14 days of development on what is called the “pre-embryo” (Franklin 2013; Warnock 1985; Mulkay 1997; Bigg 2017) as well as of other frames across the world (Jasanoff 2005; Bharadwaj 2012; Hurlbut 2017; Thompson 2013). Moreover, the reference to the “couple” implies a very specific set of norms that are stressed in the Public Health Code (articles L2141-1 and L2141-2). Patients have to be heterosexual, within a specified procreative age, and be married / have been a couple for at least two years. Again, this is very specific to France and a lot of countries have opened assisted reproductive technologies to single persons and/or same sex couples. In Israel, for example, single women can access fertility treatments and it is perfectly conceivable that the embryo used to derive the I6 line was gifted by someone who was single.

Interestingly, the authorization to import a huES line, given by the National Biomedical Agency, based on the above law articles, is neither the recognition of other bioethical frames nor a complete recognition of a standardized global material for research. The authorization is a process of nationalization that reinvents the cells as French by framing their importation in specific words. The Civil Code's ontological vocabulary has no reference to a sociological reality outside of France. These words can be seen as drivers for new meanings. When cells enter the French territory, they are inscribed in a set of institutional practices that give them new meaning relating to French history, bioethics and legislation. This ontological production is not only discursive but has very practical consequences.

The Israel number 6 cell line was nationalized by regulating laboratory biobank management. Even if the process of derivation has completely transformed cells which are no longer embryos, even if the cells come from a different country with a different history, economy and religion, these cells gain a status of specialness as soon as they enter French territory. An inspector of the National Biomedical Agency commented that their traceability must be “total.” Penal rules are very clear, and anyone who breaks these rules risks a fine of up to 30 000 euros and two years of imprisonment (article L2163-7 of public health Code and article 511-19-2 of penal Code). “It is a very delicate context relating to the Human and human reproduction,” commented the inspector during an interview with Merleau-Ponty (28th of July 2015). This sentence tightly binds stem cells to their IVF past. It also stresses the delicate balance between research goals and French representations on human embryos.

Cells’ traceability can be established in several ways. For example, experiments using huES must be written in special laboratory notebooks, while all other experiments with different materials, human and non-human, can be written in the same notebook. Storage procedures must be clearly identified, and laboratories are required to provide maps of their facilities and clear identification of where the cells are stored. Whilst special incubators for cell culture are not mandatory, the basic science laboratory which cultured the I6 did so in a specific one, as the laboratory space dedicated to cell culture was big enough. Similarly, the huES bank, the physical tank, was in a special room away from all the other cells, both human and animal. Moreover, when the inspector narrated how he inspects a laboratory, he stressed that he does not look at the other cells’ management (either human or other animals) but concentrates all his attention on the huES lines. Once within French territory these cells are connected to a new ontological apparatus which inscribes them in a context that has nothing to do with personal biographies or Israeli layers, but which relates to French bioethics.

## Discussion

When the press release referred to the French clinical trial as having used “human embryonic stem cells,” it certainly enacted a “disentangled” version of stem cell science from reproductive dynamics (Glasner 2005; Gottweiss, Salter, and Waldby 2009). However, when analyzing where the cells originated from, reproduction comes back to the forefront, not as a personal biographical, but as a repronationalist dynamic. Repronationalism, in its variegated shapes and forms, underlines how the rise of stem cell science is part of a country's national identity and political economy, shaped through reproductive policies and practices (Yuval-Davis 1998; Zanini 2011; Nahman 2013; Franklin and Inhorn 2016; Roux and Courduriès 2017; Wahlberg 2018).

Furthermore, looking at which cells were used also opens up a history of travel across two national frameworks, the French and the Israeli one. Following transnational connections of biobanks from France to Israel and back, we have delineated how the cells are co-produced within different repronational contexts while circulating across the world. If the derivation of a human embryonic stem cell line implies a biological transformation of the human embryo, as well as the use of these cells in protocols which have little to do with reproduction, this article has shown that the reproductive framings of these cells inform their regenerative future. Moreover, the global circulation of these cells is not a disappearance of local contexts which are, on the contrary very much involved in the transnational logics.

The French basic science laboratory that started its research in 2005 relied on international collaborations as French bioethics and regulations had prevented the production of stem cell lines based on a specific reproductive understanding of embryos as “potential human persons.” The cells used in the scientific protocols came from Israel. The embryo that was given to produce the stem cell line was transformed into a biological material named “Israel number 6” in a context where numerous supernumerary embryos were available to donation and stem cell science associated with bioethical leniency and academic entrepreneurialism. The international character of the I6 stem cell line is an entanglement of national contexts (the French and the Israeli one) in which reproduction plays a central role, albeit in different ways. The production and circulation of the line from one country to the other materializes through the usage of several types of conservation sites, which accompany the passaging of the cells.

The meaning of passage refers not only to the ways in which scientists build networks and solve problems (Callon 1986), but also to the ways in which scientific practices of stem cell biology are co-produced (Jasanoff 2004) by repronational histories (Franklin and Inhorn 2016). The notion of passage highlights the intertwining of these different aspects in the storage of cells in several biobanks. The absence of cells in 2005 in France can be viewed as a passage as it results from the strong link between embryos, reproduction and French bioethics that views biologization as a threat to humanization. The importation of cells from Israel to France enacts co-productive dynamics that connect “the micro-worlds of scientific practice and the macro-categories of political and social thought” (*Id.*, 18). When biologists tick boxes on the French importation permits, they connect cells to French reproductive regulations that have little to do with the Israeli IVF-stem cell interface that co-produced the cells. When the inspector of the National Biomedical Agency explains how he visits stem cells labs and verifies notebooks and biobank management, he justifies his practice by referring to “the Human and human reproduction,” reproducing a French nationalized version of reproduction in the stem cell lab. Each cell passage within biology practices is the reproduction of sameness and difference, as the cells divide into two versions of themselves and microDNA changes might occur. Passaging cells across sites, institutions and countries is about circulating a common material for research (the I6 line) while modifying its understanding in two different repronational contexts and a global market.

This focus on long-term genealogy contributes to the ongoing critique of the bench to bedside view of bioscientific translation (Wainwright *et al*. 2006; Cribb *et al*. 2008; Martin, Brown, and Kraft 2008; Jent 2017). By researching what makes basic research practices possible, local understandings of reproduction and national histories of science and technology arise. Within global dynamics, bioscientists deal with national frameworks which have strong stances on what embryos and their stem cells are, as well as the reconfigurations involved in passaging materials from one country to the other.

## Notes

1. http://presse.inserm.fr/therapie-cellulaire-de-linsuffisance-cardiaque-premiere-implantation-de-cellules-cardiaques-derivees-de-cellules-souches-embryonnaires-humaines/17502/

2. In this article, the term “biobank” is used with a wide definition that is not restricted to a definition stemming from national and international regulations. First of all, this text builds on the context of the early 2000's when such regulations were still in the making. In 2005, in the huES world, the only institutionalized biobank was the UK stem cell bank from Glyn Stacey. But, huES lines were nonetheless derived, stored and circulated across countries. In this text, we use the definition of Milanovic, Pontille, and Cambon-Thomsen (2007, 3). When referring to biological samples they write that
there is no consensus on terminology in regard to these resources, which are designated by diverse terms: collections, biological resource centres, biological databases, biorepositories, biobanks, to name only a few. ‘Biobank’ will be the term used throughout this paper for such organised collections. (…) A biobank can be generically defined as a group of biological samples that may consist of any tissue or fluid containing nucleated cells (solid tissues, blood, saliva, etc.) that are associated with computerized files generally including the origin of the donors, clinical data and biological data. For example, Michel Pucéat's laboratory, which imported and stored the I6 cells was not strictly speaking a biobank but a “site of conservation” with authorizations from the Agency of Biomedicine to import the cells, store them and research with them. The origin of donors is anonymous in that case, and the clinical data are unknown apart from negative virology. As a “site of conservation,” the laboratory has to respect some rules about where the cells are stored in the facility, in a certain kind of room and container, using a certain kind of cell registry. These rules are not as plentiful as the ones used to regulate and legally define “biobanks” in a hospital or what is called in France “clinical investigation centers” where the cells that are banked may be used for cell transplantation or just genetic screening or, eventually, for research in academic labs.

3. The word “circulation” is a tribute to a conference were an early version of this paper was presented. *Biocircularities: Lives, times and technologies*, organized by Branwyn Poleykett and Karen Jent, CRASSH, University of Cambridge. 31-03-2017/01-04-2017

4. Franklin and Inhorn (2016) introduced the term repronationalism to describe “how each country uses reproductive policy to shape its national identity, while similarly expressing its national identity through its reproductive policy” (Also see, Franklin (2016).

5. There are no national statistics on what happens with supernumerary embryos after being stored for five years. Yet, according to recent finding by Raz et al. (2016) the donation rate for research is very low in Israel.

6. The number of IVF cycles in Israel totaled 34,538 in 2010 compared to nearly 32,000 during 2014 and 18,000 in 2000. (http://www.jpost.com/Health-and-Science/Number-of-IVF-births-in-Israel-risesin-last-decade).
